# Sex-specific association between alcohol consumption and liver cirrhosis: An updated systematic review and meta-analysis

**DOI:** 10.3389/fgstr.2022.1005729

**Published:** 2022-10-13

**Authors:** Laura Llamosas-Falcón, Charlotte Probst, Charlotte Buckley, Huan Jiang, Aurélie M. Lasserre, Klajdi Puka, Alexander Tran, Jürgen Rehm

**Affiliations:** ^1^ Institute for Mental Health Policy Research, Centre for Addiction and Mental Health, Toronto, ON, Canada; ^2^ Heidelberg Institute of Global Health, Medical Faculty and University Hospital, Heidelberg, Germany; ^3^ Campbell Family Mental Health Research Institute, Centre for Addiction and Mental Health, Toronto, ON, Canada; ^4^ Department of Psychiatry, University of Toronto, Toronto, ON, Canada; ^5^ Department of Automatic Control and Systems Engineering, University of Sheffield, Sheffield, United Kingdom; ^6^ Dalla Lana School of Public Health, University of Toronto, Toronto, ON, Canada; ^7^ Addiction Medicine, Department of Psychiatry, Lausanne University Hospital, Lausanne, Switzerland; ^8^ Department of Epidemiology and Biostatistics, Western University, London, ON, Canada; ^9^ Institute of Clinical Psychology and Psychotherapy & Center of Clinical Epidemiology and Longitudinal Studies (CELOS), Technische Universität Dresden, Dresden, Germany; ^10^ Center for Interdisciplinary Addiction Research (ZIS), Department of Psychiatry and Psychotherapy, University Medical Center Hamburg-Eppendorf (UKE), Hamburg, Germany; ^11^ Faculty of Medicine, Institute of Medical Science, University of Toronto, Toronto, ON, Canada; ^12^ Department of International Health Projects, Institute for Leadership and Health Management, I.M. Sechenov First Moscow State Medical University, Moscow, Russian Federation, Russia

**Keywords:** liver cirrhosis, alcohol, systematic review, meta-analysis, epidemiology

## Abstract

Different studies have shown that females develop liver diseases at lower levels of alcohol consumption than males. Our aim was to quantify the dose-response relationship between alcohol consumption and the risk of liver cirrhosis by sex and identify the differences between females and males. A systematic review was conducted using PubMed/Medline and Embase to identify longitudinal and case-control studies that analyzed the relationship between the level of alcohol use and liver cirrhosis (LC) incidence, and mortality (ICD-8 and ICD-9 codes 571 and ICD-10 codes K70, K73, K74). Pooled relative risks (RR) were calculated by random effects models. Restricted cubic splines were used to model the dose-response relationship. A total of 24 studies were included in the analysis. There were collectively 2,112,476 females and 924,853 males, and a total of 4,301 and 4,231 cases of LC for females and males, respectively. We identified a non-linear dose-response relationship. Females showed a higher risk for LC compared to males with the same amount of alcohol consumed daily. For instance, drinking 40 g/day showed RRs of 9.35 (95% CI 7.64-11.45) in females and 2.82 (95% CI 2.53-3.14) in males, while drinking 80 g/day presented RRs of 23.32 (95% CI 18.24-29.82) in females and 7.93 (95% CI 7.12-8.83) in males. Additional analyses showed that a higher risk for females was found for morbidity and for mortality. Understanding the influence of sex on the association of alcohol consumption and the risk of LC is needed to develop recommendations and clinical guidelines for prevention and treatment.

**Systematic Review Registration:**

https://www.crd.york.ac.uk/prospero/display_record.php?ID=CRD42022299680, identifier CRD42022299680.

## Introduction

Globally, the incidence of liver cirrhosis (LC) has shown an increasing trend in females and a stabilized trend in males in recent years (1), with certain areas such as the Eastern Mediterranean Region, African Region and South-East Asia, having an increasing trend of LC in females and males. Other areas had an increasing trend only in males, such as the European Region, and only in females, such as the Region of the Americas. In addition, although death and disability-adjusted life years (DALY) global rates of LC decreased from 1990 to 2019, the number of deaths and DALYs and the proportion of all global deaths due to LC have increased (2), being ranked the 8th most common non-communicable disease cause of death globally in 2019 (1). With alcohol use being a key risk factor in the progression and mortality of LC (3), particularly among females (4, 5), efforts should be made to develop alcohol control policies and prevention measures to address this concerning public health problem.

There is evidence that a dose-response relationship between alcohol consumption and LC exists (6–8) and, at the same level of alcohol consumption, females seem to have a higher risk of developing LC than males (7). By studying how the dose-response relationship varies by cause of LC and by fatal vs non-fatal events in females and males (9), we will have strong evidence to inform policy makers and prevention experts on whether most of their efforts should be directed to the general population level or targeted towards females and males by their levels of alcohol use. For instance, if the risk curves are more linear, a general population approach will yield better results, whereas with more exponential risk curves targeted approaches should be recommended (10). Consequently, there is a need to have global, updated, and precise risk curves on the association of alcohol consumption and LC in females and males from which we can identify the meaningful risk increases for each sex. Accordingly, our aim was to quantify the dose-response relationship on alcohol use and the risk of LC by sex using meta-analysis and meta-regression models, and to identify the differences between females and males in the risks of morbidity and mortality due to LC.

## Materials and methods

### Systematic review

We conducted a systematic review using PubMed/Medline and Embase databases from their inception to October 12, 2021, applying the Preferred Reporting Items for Systematic Reviews and Meta-Analyses (PRISMA) criteria (see PRISMA checklist (11) in the **Supplementary Material 1**). An updated search was conducted on January 14, 2022 with no new references discovered. Any combination of key words and MeSH terms relating to alcohol consumption, LC, and observational studies were included (for the complete search strategy, see **Supplementary Material 2**) and the reference list of relevant articles was reviewed. All references were screened by one author, with independent verification by two additional reviewers. We screened the publications which resulted from a wider review on alcohol consumption and LC (PROSPERO registration number CRD42022299680) in order to identify papers with sex-specific results.

Studies included were articles with a longitudinal or case control design. The exposure variable was the quantity of alcohol use. The outcome was LC morbidity (incidence of LC or decompensated LC) and mortality (ICD-8 and ICD-9 codes 571 and ICD-10 codes K70, K73, K74). Studies were excluded if they were not published as full reports, they used a cross sectional design, or there was not enough data to compute the relative risk related to alcohol use. For this review, we also excluded articles which presented their results with both sexes combined.

### Data extraction

Two authors extracted relevant information using a standardized spreadsheet which included the title, first author, year of publication, country, study design, year of study at baseline and follow-up, sex, sample size, cause of LC, socioeconomic status, alcohol consumption categories, time period of alcohol consumption, risk estimates (relative risk (RR), odds ratio (OR) and hazard ratio (HR)) with their corresponding 95% CIs, adjustments, and outcomes.

If the quantity of alcohol use was not presented in grams per day, we converted it based on the size of a standard drink in the country the study was based on. When alcohol was given in ranges, the midpoint was taken. If there was no upper bound for the highest category, 75% of the width from the previous category range was taken and added to the lower bound, and this value was used to represent the highest level of alcohol use.

### Quality assessment

We used an adapted version of the Cochrane Risk-of-Bias Tool for Non-Randomized Studies (ROBINS-I) (12) to assess the risk of bias in the studies included in our analysis (for details on the adaptation, see **Supplementary Material 3**). The evidence was rated based on the Grading of Recommendations Assessment, Development and Evaluation approach (13). Each article was rated by at least two authors and several consensus conferences were held to produce the final ratings. The overall bias for each study was rated with one of the following scores: low, moderate, serious, or critical, with the ranking going from the lowest to the highest risk of bias.

### Statistical analysis

We harmonized the reference categories of all the included studies to have lifetime abstainers serve as the reference category. This step was necessary to avoid the “sick quitter bias” which is the bias of including abstainers who quit drinking for health reasons (14) (for similar analyses, see (15)). RRs were pooled with the inverse-variance method using Restricted Maximum Likelihood (REML) random effects model (16). Heterogeneity was quantified using the Cochrane Q-test and the I^2^ statistic. In order to obtain the dose-response curves, five models were tested (linear, quadratic, restrictive cubic splines, cubic polynomial, and fractional polynomial) (17). The model which best fit our data was selected based on Akaike Information Criterion (AIC) and Bayesian information criterion (BIC) statistics.

We ran meta-regression models, integrating sex, cause of LC, quality score, and outcomes to test their interactions with the amount of alcohol consumed. The differences between the estimates were tested using a Wald-type test. In addition, we conducted a sensitivity analysis excluding the study conducted by Liu and colleges (18), as it had the highest weight in our analysis due to its large sample size. All analysis were conducted using meta (19) and metafor (20) packages in R software version 4.2.1 (21).

## Results

**Figure 1:**
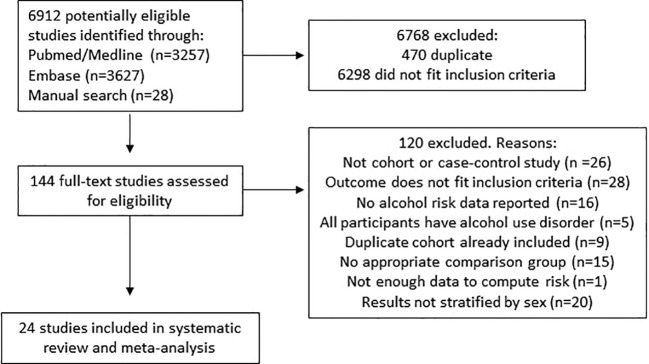
Flow-chart of the study selection process.

A total of 24 studies were included in the analysis (**Figure 1**); 13 of the studies provided results for females and 19 provided results for males. **Table 1** presents the studies’ characteristics. There were collectively 2,112,476 females and 924,853 males, and a total of 4,301 and 4,231 cases of LC for females and males respectively. The majority of studies were cohort studies (67%). In total, 10 studies (42%) provided results for LC morbidity, 11 studies (46%) were mortality studies, and three studies (12%) presented their results with both morbidity and mortality combined. As for the type of LC, 20 studies provided data including all causes of LC, two studies for alcohol-related LC, one study for HCV-induced LC, and one study for alcohol-related LC and HCV-induced LC combined. In the risk-of-bias assessment, 10 studies had a moderate score, six studies had a serious score, and eight studies had a critical score.

**Table 1:** Characteristics of the included studies.

Authors	Year	Country	Study design	FU- years	N	Female (%)	Cause LC	Alcohol use in g/day: n	Relative risk*	CI 95% lower	CI 95% upper	Adjusted		Outcome		Quality score
														Category	P (%)
Alemy-Carreau et al. (22)	1996	France	Case-control	N.A.	221	0	ALD + HCV	0 g/d: 24	Reference	–	–	No		Cirrhosis	101 (46%)	Critical
								100 g/d: 197	121.1	49.4	298.9				
Askgaard et al. (23)	2015	Denmark	Cohort	15	55,917	52	ALD	Male				Yes		Cirrhosis and LC mortality	257 (1%)	Serious
								0-24 g/d: 15,028	Reference	–	–				
								24-48 g/d: 6,800	2.33	1.52	3.58				
								48-72 g/d: 2,774	6.98	4.65	10.5				
								72-96 g/d: 1,062	13.12	8.51	20.23				
								96-120 g/d: 445	29.03	18.63	45.24				
								>120 g/d: 174	50	30.12	82.96				
								Female							85 (0.3%)
								0-24 g/d: 23,278	Reference	–	–			
								24-48 g/d: 4,242	3.49	2	6.12			
								48-72 g/d: 744	16.2	9.16	28.7			
								>72 g/d: 222	21.57	10.38	44.84			
Batey et al. (24)	1992	Australia	Case-control	N.A.	158	0	All cause	≤40 g/d: 109	Reference	–	–	No		Cirrhosis	43 (27%)	Serious
								41-80 g/d:23	8.8	3.2	24.3				
								>80 g/d: 26	21.9	7.7	62.9				
Becker et al. (25)	2002	Denmark	Cohort	5	30,630	47	ALD	Male				Yes		Cirrhosis and LC mortality	212 (1%)	Moderate
								<1.7 g/d	7.76	3.35	18				
								1.7-12 g/d	Reference	–	–				
								12-36 g/d	2.34	1.18	4.62				
								36-60 g/d	1.34	0.7	2.56				
								>60 g/d	2.63	1.39	5				
								Female							80 (0.6%)
								<1.7 g/d	1.11	0.43	2.84			
								1.7-12 g/d	Reference	–	–			
								12-36 g/d	4.48	2.21	9.08			
								36-60 g/d	9.08	3.6	22.77			
								>60 g/d	11.85	3.74	37.48			
Blackwelder et al. (26)	1980	USA	Cohort	4	7888	0	All cause	0 g/d: 3,747	2.11	0.25	17.49	No		LC mortality	16 (0.2%)	Serious
								1-10 g/d: 1,316	Reference	–	–				
								11-30 g/d: 1,593	1.65	0.15	18.2				
								≥30 g/d: 1,232	7.48	0.92	60.69				
Boffetta et al. (27)	1990	USA	Cohort	12	276,802	0	All cause	0 g/d: 153,043	Reference	–	–	Yes		LC mortality	687 (0.2%)	Moderate
								14 g/d: 33,229	1.21	0.86	1.69				
								28 g/d: 23,558	3.15	2.39	4.16				
								42 g/d: 11,257	5.39	4	7.26				
								56 g/d: 7,309	8.67	6.45	11.6				
								70 g/d: 3,368	10.6	7.36	15.3				
								≥84 g/d: 7,698	18.1	14.1	23.2				
Corrao et al. (28)	1993	Italy	Case-control	N.A.	640	35	All cause	Male				Yes		Cirrhosis	207 (50%)	Serious
								LTA: 28	Reference	–	–				
								25 or 50 g/d: 58	1.4	0.6	3.1				
								75 or 100 g/d: 98	1.6	0.7	3.8				
								125 or 150 g/d: 95	2.1	0.9	4.7				
								≥175 g/d: 135	4.9	2.2	10.9				
								Female							113 (50%)
								LTA: 62	Reference	–	–			
								25 or 50 g/d: 82	0.5	0.2	0.8			
								75 or 100 g/d: 38	1.4	0.6	3.2			
								125 or 150 g/d: 26	2.6	1	6.5			
								≥175 g/d: 18	8.7	2.3	33			
Corrao et al. (29)	1997	Italy	Case-control	N.A.	1,113	41	All cause	Male				Yes		Cirrhosis	300 (46%)	Serious
								LTA: 48	Reference	–	–				
								<50 g/d: 255	1.9	0.62	5.8				
								50-100 g/d: 132	9.1	2.94	28.18				
								≥100 g/d: 220	31.4	10.3	95.76				
								Female							162 (35%)
								LTA: 104	Reference	–	–			
								<50 g/d: 306	2.09	0.69	6.3			
								50-100 g/d: 35	7.5	3.46	16.25			
								≥100 g/d: 13	20.36	6.3	65.79			
Fuchs et al. (30)	1995	USA	Cohort	12	85,709	100	All cause	0 g/d: 25535	4.76	1.66	39.29	Yes		LC mortality	52 (0.06%)	Moderate
								0.1-1.4 g/d: 11304	Reference	–	–				
								1.5-4.9 g/d: 18460	3.29	1.14	9.43				
								5-14.9 g/d: 17783	6.05	2.57	14.33				
								15-29.9 g/d: 8106	8.86	3.62	21.86				
								≥30 g/d: 4521	12.14	5.05	29.1				
Garfinkel et al. (31)	1988	USA	Cohort	12	581,321	100	All cause	LTA: 467,382	Reference	–	–	No		LC mortality	589 (0.01%)	Critical
								14 g/d: 20,000	2.46	1.87	3.25				
								28 g/d:13,000	7.4	5.9	9.27				
								49 g/d: 10,000	14.21	10.77	18.75				
								77 g/d: 12,000	16.7	12.1	23.04				
								≥84 g/d: 2,000	28.29	20.21	39.59				
Gordon et al. (32)	1984	USA	Cohort	22	4747	56	All cause	Male				No		LC mortality	14 (0.7%)	Critical
								0 g/d: 402	Reference	–	–				
								0.7-20 g/d: 1107	1.45	0.16	12.96				
								21-41 g/d: 344	3.51	0.37	33.55				
								41-61 g/d: 131	9.21	0.97	87.75				
								62-82 g/d: 50	8.04	0.51	126.55				
								83-248 g/d: 72	11.17	1.03	121.55				
								Female							
								0-7-20 g/d: 2434	Reference	–	–			
								21-61 g/d: 191	1.59	0.2	12.67			
								62 g/d-248 g/d: 16	19	2.52	143.34			
Gordon et al. (33)	1987	USA	Cohort	28	1,762	0	All cause	0 g/d: 585	Reference	–	–	No		LC mortality	15 (0.9%)	Critical
								0.7-20 g/d: 842	0.52	0.12	2.32				
								21-41 g/d: 175	2.51	0.57	11.1				
								41-61 g//d: 100	2.93	0.54	15.76				
								> 62 g/d: 60	7.31	1.68	31.91				
Im et al. (34)	2021	China	Cohort	10.1	218,341	59	All cause	Male				Yes		Cirrhosis	1210 (1%)	Moderate
								1 g/d: 117,072	Reference	–	–				
								<20 g/d: 24,171	1.29	1.06	1.57				
								20-40 g/d: 18,182	2.47	2.1	2.91				
								40-60 g/d: 12,318	3.13	2.59	3.79				
								≥ 60 g/d: 12,318	8.15	7.04	9.43				
								Female							946 (3%)	
								1 g/d: 28,396	Reference	–	–				
								16 g/d: 5,896	1.09	0.71	1.68				
Khan et al. (35)	2000	Japan	Case-control	N.A.	106	0	HCV	0 g/d: 40	Reference	–	–	No		Cirrhosis	54 (51%)	Critical
								<80 g/d: 42	6	2.29	15.69				
								>80 g/d: 24	6	1.98	18.21				
Klatsky et al. (36)	2003	USA	Cohort	20	128,934	56	All cause	Male				No		LC mortality	146 (0.3%)	Moderate
								LTA: 4,125	Reference	–	–				
								<0.5 g/d: 8,105	0.7	0.32	1.53				
								<14 g/d: 21,264	0.5	0.25	1.02				
								14-28 g/d: 13,512	1.3	0.68	2.48				
								42-70 g/d: 5,905	3.3	1.7	6.4				
								≥84 g/d: 1,535	8.3	3.97	17.34				
								Female							86 (0.1%)
								LTA: 11,373	Reference	–	–			
								<0.5 g/d: 19,312	1.2	0.42	3.42			
								<14 g/d: 26,631	2.5	1	6.26			
								14-28 g/d: 9,896	4.7	2.02	10.95			
								42-70 g/d: 2,523	14.2	5.94	33.96			
								≥84 g/d: 469	15.2	5.81	39.76			
Kono et al. (37)	1986	Japan	Cohort	18	5,135	0	All cause	LTA: 1,074	Reference	–	–	Yes		LC mortality	43 (0.8%)	Moderate
								<27 g/d: 1,034	0.3	0.1	1.1				
								>27 g/d: 925	1.8	0.8	4				
Liu et al. (18)	2009	UK	Cohort	6	1,290,413	100	All cause	0 g/d: 305,652	1.41	1.23	1.61	Yes		Cirrhosis and LC mortality	2105 (0.2%)	Moderate
								1-2 g/d:372,065	Reference	–	–				
								3-7 g/d:294,353	1.24	1.08	1.44				
								8-16 g/d: 241,307	1.84	1.6	2.11				
								≥17 g/d: 67,360	4.32	3.71	5.03				
Norton et al. (38)	1987	Australia	Case-control	N.A.	135	100	All cause	<40 g/d: 102	Reference	–	–	No		Cirrhosis	36 (27%)	Critical
								>40 g/d: 33	784	84.52	7272.06				
Pequignot et al. (39)	1978	France	Case-control	N.A.	962	0	All cause	0-20 g/d: 188	Reference	–	–	Yes		Cirrhosis	184 (19%)	Serious
								21-40 g/d: 222	3.1	0.84	11.42				
								41-60 g/d: 180	6.2	1.76	21.83				
								61-80 g/d: 132	13.8	4.06	46.92				
								81-100 g/d: 88	29.6	8.71	100.55				
								101-120 g/d: 54	41	11.61	144.83				
								121-140 g/d: 38	124.3	33.1	466.75				
								>141 g/d: 60	659.3	159.57	2723.98				
Schult et al. (40)	2017	Sweden	Cohort	33	1,462	100	All cause	0 g/d	Reference	–	–	No		Cirrhosis	11 (0.8%)	Critical
								10 g/d	2.16	1.57	2.99				
								30 g/d	10.08	3.87	26.73				
Stroffolini et al. (41)	2010	Italy	Case-control	N.A.	397	34	All cause	Male				No		Cirrhosis	111 (14%)	Critical
								12-24 g/d: 215	Reference	–	–				
								36 g/d: 56	1.2	0.5	2.9				
								≥36 g/d: 239	4.3	2.5	7.3				
								Female							26 (3%)
								12-24 g/d: 204	Reference	–	–			
								36 g/d: 18	0.8	0.1	6.5			
								≥36 g/d: 37	5.7	2.3	14.5			
Yang et al. (42)	2012	China	Cohort	15	218,189	0	ALD	0 g/d: 145,323	Reference	–	–	Yes		LC mortality	418 (0.2%)	Moderate
								<20 g/d: 14,208	1.12	0.76	1.64				
								20-40 g/d: 19,391	0.84	0.59	1.21				
								40-60 g/d: 18,681	1.16	0.85	1.56				
								60-100 g/d: 10,870	1.22	0.83	1.79				
								≥100 g/d: 9,716	1.74	1.22	2.48				
Yi et al. (43)	2016	Korea	Cohort	6	107,735	0	ALD	<1.3 g/d: 34,435	Reference	–	–	Yes		LC mortality	178 (0.2%)	Moderate
								1.3-8 g/d: 31,341	1.32	0.81	2.18				
								9-17 g/d: 15,162	2.45	1.48	4.07				
								18-35 g/d: 15,764	2.77	1.71	4.5				
								≥36 g/d: 11,033	4.03	2.51	6.46				
Yuan et al. (44)	1997	China	Cohort	9	18,244	0	ALD	LTA: 10,471	Reference	–	–	Yes		LC mortality	35 (0.2%)	Moderate
								1.7-48 g/d: 6,189	0.46	0.17	1.22				
								≥50 g/d: 1,201	2.99	1.12	7.94				

FU, follow-up; N, sample size; LTA, lifetime abstainers; CI, confidence intervals; P, percent in sample; N.A., not applicable; ALD, alcohol-related liver disease; HCV, hepatitis C virus; LC, liver cirrhosis. *Risk-relation estimates were based on either odds ratios, relative risks, or hazard ratios.

**Figure 2:**
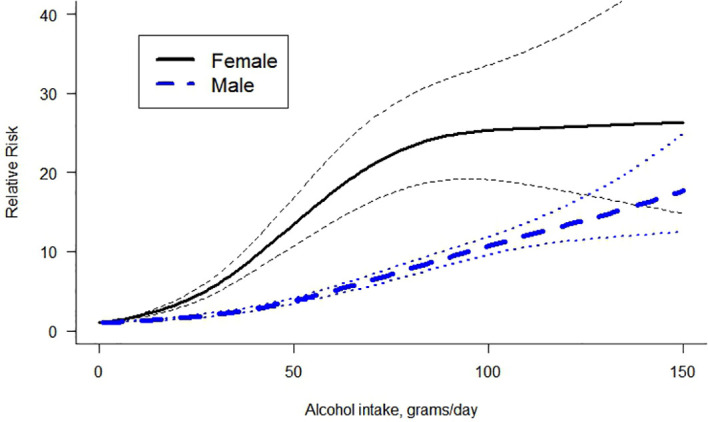
Dose-response curve between alcohol intake in grams per day and the risk of liver cirrhosis by sex.

A non-linear dose-response curve was identified for both females and males. The restrictive cubic spline model (17) was the model that best fit our data (for reasoning on the selection of the model see **Supplementary Material 4**). For the same amount of alcohol use, females presented a higher relative risk of LC than males (**Figure 2**). For both curves, the risk of LC increased with the average number of grams of alcohol consumed daily. **Table 2** presents the RRs and the risk ratios of females vs males. The risk ratio was not constant, first increasing in value and then decreasing as the level of alcohol consumption became higher.

**Table 2:** Relative risk and risk ratio by sex for some levels of alcohol use.

Grams of pure alcohol per day	Female RR (95% CI)	Male RR (95% CI)	Risk Ratio
20	3.34 (2.81 - 3.97)	1.60 (1.46 - 1.77)	2.09
40	9.35 (7.64 - 11.45)	2.82 (2.53 - 3.14)	3.32
60	17.54 (13.80 - 22.91)	5.09 (4.85 - 5.65)	3.45
80	23.32 (18.24 - 29.82)	7.93 (7.12 - 8.83)	2.94
100	25.33 (19.09 - 33.60)	10.76 (9.69 - 11.94)	2.35
120	25.77 (17.62 - 37.68)	13.40 (11.30 - 15.88)	1.92

We identified several statistically significant differences in the overall meta-regression model (for full model results and estimates, see **Supplementary Material 5**). We obtained a significantly higher LC risk for females than for males (beta=-0.0319, se=0.00407; estimate for the interaction between sex and dose of alcohol consumption with female as reference). In addition, mortality studies presented a significantly higher risk compared to morbidity studies. Based on the quality scores obtained in the risk assessment, studies with serious and critical scores presented a higher risk compared to studies with moderate scores. Finally, we identified a significant steeper slope in HCV-related studies compared to all-cause LC and a steeper slope in all-cause LC compared to alcohol-related LC, this latter not being statistically significant.

**Figure 3:**
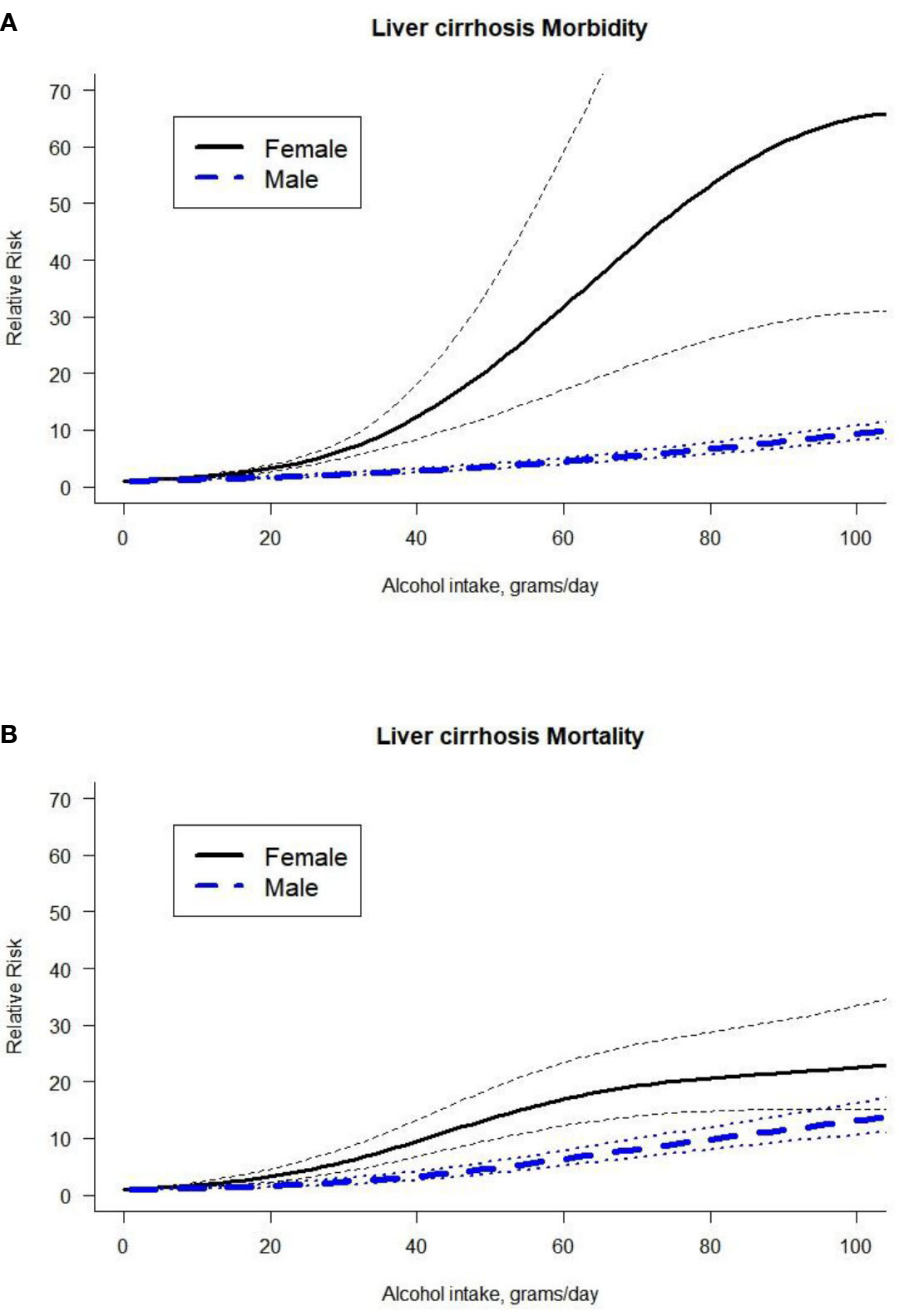
Dose-response curve between alcohol intake in grams per day and the risk of liver cirrhosis by outcome (**A**. Liver cirrhosis morbidity, **B**. Liver cirrhosis mortality) and by sex.

In addition, we identified a higher risk for morbidity outcomes in females compared to males and a significantly higher risk for mortality due to LC in females compared to males (**Figure 3**, for full model results, see **Supplementary Material 6**). We also tested the interaction of morbidity and mortality stratified by sex (for full model results, see **Supplementary Material 7**). In females, we did not identify statistically significant differences between morbidity studies and mortality studies. For males, mortality studies presented a significantly higher risk compared to morbidity studies.

In our sensitivity analysis, we excluded the study by Liu and colleges (18) which provided four estimate points with the corresponding weights: 14%, 14%, 15%, 13% in the restrictive cubic splines model in the overall dose-response relationship for females. When we ran the analysis with the study excluded, the shape of the curve attenuated but there were still statistically significant differences compared to males (see **Supplementary Material 8**) and there were no changes in the significance and direction of the regression weights for the other variables studied.

## Discussion

Differences in the risk of LC conditional on alcohol use between females and males have been identified, showing that, for females, the same level of alcohol consumed presents a higher risk of LC than for males as well as a higher mortality risk of LC, irrespective of the cause of LC. Our study is the most comprehensive and presents the most up to date estimates currently available. These results are consistent with previous reviews published (6, 7), which identified an increasing dose-response relationship in females. Our review identified six additional studies for females and 13 additional studies for males compared to the most recent review (7) mainly due to the inclusion of additional biological pathways of LC. In addition, it was not restricted to studies of all causes of LC combined, and thus we had the necessary power to study the impact of other variables. Importantly, the dose-response curves for females accelerate quickly, characterized by a non-linear curve with a more exponential relationship before flattening out.

The basis for this difference may partly be explained by differences in alcohol pharmacokinetics and pharmacodynamics between females and males (45, 46). Females generally have a relatively lower total water content and are generally smaller than males, resulting in higher blood alcohol concentrations for the same amount of ingested alcohol. Frezza et al. (47), confirmed this theory and identified that females also present a lower rate of alcohol dehydrogenase activity in the gastric mucosa, consequently producing a higher bioavailability of alcohol when lower doses of alcohol are consumed and a longer time period during which these levels are elevated. Thus, the activity of gastric metabolism is decreased in dependent alcohol use and sex differences are less apparent in heavy drinkers (48). In addition, estrogens are involved in alcohol-related liver damage by increasing the susceptibility of Kupffer cells to endotoxins (49). When activated, Kupffer cells release cytokines and produce hepatic inflammation, playing a role in hepatotoxicity after alcohol exposure (50). In contrast, when estrogen is blocked in animal models, the alcohol-related injury is attenuated (51). Therefore, Kupffer cells are an additional key element in the sex differences in liver injury caused by alcohol use.

The gender-gap that historically existed in the level of alcohol consumption between men and women is narrowing, with women increasing their consumption, including a notable increase in high-risk drinking (52). This phenomenon is a concerning public health problem which can consequently lead to adverse health effects. Additionally, gender-related differences in alcohol use regarding drinking patterns and problematic drinking (53), have been decreasing as the newest generations are being exposed to a social, economic and cultural homogenization (54). Knowing the unique risk that alcohol has for women, increases in their consumption are concerning and efforts should be directed to build awareness for this issue. More specifically for LC, prevention campaigns should take into consideration that females incur more than twofold risks of LC compared to males at all levels of alcohol consumption, with the excess risk of females being highest at 60 grams of pure alcohol per day.

In addition, given the quick acceleration of risk curves in females, targeted approaches directed to females in the health-care system seem promising. This is especially true with the apparent gender-gap in today’s clinical practice, since women are not routinely asked about their alcohol use (4, 5). In general, alcohol assessment during healthcare visits is key to the early identification of harmful alcohol use as well as for quickly conducting brief interventions or referrals for specialty treatment. Brief interventions and alcohol use disorder treatment have been shown to be effective and relatively cost effective in decreasing consumption (55, 56), and thus in reducing the risk for LC. One study reported that men have a higher rate of accessing healthcare prior to being diagnosed with alcohol-related LC than women, but they are less likely to receive interventions or referrals than women (57). Nevertheless, women report having less social support for treatment engagement when dealing with alcohol use disorders (58). To formulate effective and accessible prevention and treatment options, these factors should be taken into account.

There are some limitations to our study that should be considered. First, we performed a meta-analysis based on an underlying literature search. Therefore, the evidence is limited to the research available and there is a longstanding and consistent history of poor measurement of alcohol use in epidemiological studies due to social desirability bias (underreporting) resulting from survey data. Also, there was a lack of data regarding the causes of LC, which leads us to be cautious when interpreting the differences identified between underlying causes of LC. Nevertheless, the same differences were identified in a wider analysis that included studies with both sexes combined. In addition, females accounted for more than twice the subjects and cases than males, and the study conducted by Liu and colleagues (18) represented more than half of the weight in our analysis. This large prospective study had a relatively high-quality methodology and, although the dose-response curve attenuated when we excluded this study from our analysis, we did not find differences in the direction of the curves or in beta-coefficients for other variables. As for the interpretation of the risk of bias score, although more than half of our studies received a serious or critical score, this was mainly because they did not include a time-variant confounding variable, which left us without a single article with a low bias score, and many articles with scores of serious and critical bias. Finally, alcohol use is often measured by self-report, which may lead to bias, although it has been shown to be valid overall (59).

By using a systematic and standardized methodology we were able to quantify the dose-response relationships in females and males and identify the differences in both curves and according to the outcomes. The higher risk observed in females, both in the progression of and mortality due to LC, calls for action. In primary care, unless alcohol use is screened for systematically, women´s drinking tends to be overlooked (60). Early identification of excessive alcohol use should be a regular practice in health-care facilities to properly diagnose and treat at-risk individuals and prevent further progression in individuals with lower levels of consumption (61).

## Data availability statement

The original contributions presented in the study are included in the article/**Supplementary Material**. Further inquiries can be directed to the corresponding author.

## Author contributions

Conceptualization: CP and JR. Methodology: LL-F, JR, HJ and AT. Data curation: LL-F, HJ, KP, AL, CB, CP and AT. Formal analysis: LL-F, HJ and AT. Funding acquisition, CP. Investigation: LL-F, KP, AL, CB, CP and JR. Project administration: CP. Resources: CP and JR. Software: LL-F, JR, HJ and AT. Supervision: CP and JR. Validation: JR and HJ. Visualization: LL-F and AT. Writing—original draft, LL-F and JR. Writing—review and editing: all authors. All authors have read and agreed to the published version of the manuscript. All authors contributed to the article and approved the submitted version.

## Funding

Research reported in this publication was supported by the National Institute on Alcohol Abuse and Alcoholism of the National Institutes of Health under Award Numbers R01AA028009 and R01AA028224. The content is solely the responsibility of the authors and does not necessarily represent the official views of the National Institutes of Health.

## Acknowledgments

We would like to thank Ms. Astrid Otto for referencing and copyediting the text.

## Conflict of interest

Author AL reported grants from Swiss National Science Foundation (SNSF) during the conduct of the study.

The remaining authors declare that the research was conducted in the absence of any commercial or financial relationships that could be construed as a potential conflict of interest.

## Publisher’s note

All claims expressed in this article are solely those of the authors and do not necessarily represent those of their affiliated organizations, or those of the publisher, the editors and the reviewers. Any product that may be evaluated in this article, or claim that may be made by its manufacturer, is not guaranteed or endorsed by the publisher.
