# Supplementary material for: Sex-specific association between alcohol consumption and liver cirrhosis: An updated systematic review and meta-analysis
Source: Front Gastroenterol (Lausanne). 2022 Oct 13;1:1005729. doi: 10.3389/fgstr.2022.1005729 (PMC10016085; doi:10.3389/fgstr.2022.1005729)
Supplement: Supplementary file 1 [file DataSheet_1.pdf]

Supplementary material

Table of contents

Supplementary Material 1. PRISMA 2020 checklist 2
Supplementary Material 2. Search strategy 5
Supplement Material 3. Cochrane Risk-of-Bias Tool for Non-Randomized Studies (ROBINS-I) adapted – risk of bias assessment 6
Supplementary Material 4. Results of model selection 9
Supplementary Material 5. Final meta-regression model 10
Supplementary Material 6. Meta-regression model by outcome 11
Supplementary Material 7. Meta-regression model by sex 12
Supplement Material 8. Sensitivity analysis 13

Supplementary Material 1. PRISMA 2020 checklist

Section and Topic	Item #	Checklist item	Location where item is reported
**TITLE**			
Title	1	Identify the report as a systematic review.	Yes, page #1
**ABSTRACT**			
Abstract	2	See the PRISMA 2020 for Abstracts checklist.	Yes, #2
**INTRODUCTION**			
Rationale	3	Describe the rationale for the review in the context of existing knowledge.	Yes, #2,3
Objectives	4	Provide an explicit statement of the objective(s) or question(s) the review addresses.	Yes, #3
**METHODS**			
Eligibility criteria	5	Specify the inclusion and exclusion criteria for the review and how studies were grouped for the syntheses.	Yes, #3
Information sources	6	Specify all databases, registers, websites, organizations, reference lists and other sources searched or consulted to identify studies. Specify the date when each source was last searched or consulted.	Yes, #3
Search strategy	7	Present the full search strategies for all databases, registers and websites, including any filters and limits used.	Yes, Appendix 2
Selection process	8	Specify the methods used to decide whether a study met the inclusion criteria of the review, including how many reviewers screened each record and each report retrieved, whether they worked independently, and if applicable, details of automation tools used in the process.	Yes, #3,4
Data collection process	9	Specify the methods used to collect data from reports, including how many reviewers collected data from each report, whether they worked independently, any processes for obtaining or confirming data from study investigators, and if applicable, details of automation tools used in the process.	Yes, #3,4
Data items	10a	List and define all outcomes for which data were sought. Specify whether all results that were compatible with each outcome domain in each study were sought (e.g. for all measures, time points, analyses), and if not, the methods used to decide which results to collect.	Yes, #3
	10b	List and define all other variables for which data were sought (e.g. participant and intervention characteristics, funding sources). Describe any assumptions made about any missing or unclear information.	Yes, #3,4
Study risk of bias assessment	11	Specify the methods used to assess risk of bias in the included studies, including details of the tool(s) used, how many reviewers assessed each study and whether they worked independently, and if applicable, details of automation tools used in the process.	Yes, #4
Effect measures	12	Specify for each outcome the effect measure(s) (e.g. risk ratio, mean difference) used in the synthesis or presentation of results.	Yes, #3,4
Synthesis methods	13a	Describe the processes used to decide which studies were eligible for each synthesis (e.g. tabulating the study intervention characteristics and comparing against the planned groups for each synthesis (item #5)).	Yes, #4,5

Section and Topic	Item #	Checklist item	Location where item is reported
	13b	Describe any methods required to prepare the data for presentation or synthesis, such as handling of missing summary statistics, or data conversions.	Yes, #4,5
	13c	Describe any methods used to tabulate or visually display results of individual studies and syntheses.	Yes, #4,5
	13d	Describe any methods used to synthesize results and provide a rationale for the choice(s). If meta-analysis was performed, describe the model(s), method(s) to identify the presence and extent of statistical heterogeneity, and software package(s) used.	Yes, #4,5
	13e	Describe any methods used to explore possible causes of heterogeneity among study results (e.g. subgroup analysis, meta-regression).	Yes, #4,5
	13f	Describe any sensitivity analyses conducted to assess robustness of the synthesized results.	Yes, #4,5
Reporting bias assessment	14	Describe any methods used to assess risk of bias due to missing results in a synthesis (arising from reporting biases).	Yes, #4,5
Certainty assessment	15	Describe any methods used to assess certainty (or confidence) in the body of evidence for an outcome.	Not applicable
**RESULTS**			
Study selection	16a	Describe the results of the search and selection process, from the number of records identified in the search to the number of studies included in the review, ideally using a flow diagram.	Yes, #5,6, figure 1
	16b	Cite studies that might appear to meet the inclusion criteria, but which were excluded, and explain why they were excluded.	Yes, figure 1
Study characteristics	17	Cite each included study and present its characteristics.	Yes, #5 and table 1
Risk of bias in studies	18	Present assessments of risk of bias for each included study.	Yes, #5,6 and table 1
Results of individual studies	19	For all outcomes, present, for each study: (a) summary statistics for each group (where appropriate) and (b) an effect estimate and its precision (e.g. confidence/credible interval), ideally using structured tables or plots.	Yes, table 1
Results of syntheses	20a	For each synthesis, briefly summarize the characteristics and risk of bias among contributing studies.	Yes, #5,6, table 1
	20b	Present results of all statistical syntheses conducted. If meta-analysis was done, present for each the summary estimate and its precision (e.g. confidence/credible interval) and measures of statistical heterogeneity. If comparing groups, describe the direction of the effect.	Yes, #5,6
	20c	Present results of all investigations of possible causes of heterogeneity among study results.	Yes, #5,6
	20d	Present results of all sensitivity analyses conducted to assess the robustness of the synthesized results.	Yes, #5,6
Reporting biases	21	Present assessments of risk of bias due to missing results (arising from reporting biases) for each synthesis assessed.	Yes, #5,6
Certainty of evidence	22	Present assessments of certainty (or confidence) in the body of evidence for each outcome assessed.	Not applicable
**DISCUSSION**			

Section and Topic	Item #	Checklist item	Location where item is reported
Discussion	23a	Provide a general interpretation of the results in the context of other evidence.	Yes, #6-8
	23b	Discuss any limitations of the evidence included in the review.	Yes, #7
	23c	Discuss any limitations of the review processes used.	Yes, #7
	23d	Discuss implications of the results for practice, policy, and future research.	Yes, #7,8
**OTHER INFORMATION**			
Registration and protocol	24a	Provide registration information for the review, including register name and registration number, or state that the review was not registered.	Yes, #3
	24b	Indicate where the review protocol can be accessed, or state that a protocol was not prepared.	Yes, #3
	24c	Describe and explain any amendments to information provided at registration or in the protocol.	Not applicable
Support	25	Describe sources of financial or non-financial support for the review, and the role of the funders or sponsors in the review.	Yes, #8
Competing interests	26	Declare any competing interests of review authors.	Yes, #8
Availability of data, code and other materials	27	Report which of the following are publicly available and where they can be found: template data collection forms; data extracted from included studies; data used for all analyses; analytic code; any other materials used in the review.	Yes, #8

Supplementary Material 2. Search strategy

PubMed/Medline

("Case-Control Studies"[Mesh] OR "Cohort Studies"[Mesh] OR "Follow-Up Studies"[Mesh] OR "Longitudinal Studies"[Mesh]) AND (alcohol OR alcohol consumption OR "Alcohol Drinking"[Mesh] OR alcohol use disorders OR alcohol dependence OR binge drinking OR heavy drinking OR "alcohol-related disorders"[MeSH] OR alcoholism OR intoxicat* OR drunk*) AND liver cirrhosis AND (liver disease OR cirrhosis OR "Liver Cirrhosis"[Mesh] OR "Liver Cirrhosis/mortality"[Mesh]) AND ("Liver Diseases, Alcoholic"[Mesh] OR "Liver Cirrhosis, Alcoholic"[Mesh] OR hepatitis B OR HBV OR "Hepatitis B, Chronic"[Mesh] OR hepatitis C OR HCV OR "Hepatitis C, Chronic"[Mesh] OR metabolic disease OR "Metabolic Syndrome"[Mesh] OR "Non-alcoholic Fatty Liver Disease"[Mesh] OR "Hepatitis, Autoimmune"[Mesh])

EMBASE

# ▲	**Searches**
	**Study types**
1	exp Case-Control Studies/
2	exp cohort studies/ or exp follow-up studies/ or exp longitudinal studies/
3	1 or 2
	**Alcohol terms**
4	exp alcohol/exp and alcohol:kw,ab or exp alcohol consumption/ or exp alcohol drinking/
5	'alcohol dependence' or 'binge drinking' or exp heavy drinking/ or exp alcohol-related disorders/ or 'alcoholism' or exp alcoholism
6	intoxicat* or drunk*
7	4 or 5 or 6
	**Disease terms**
8	'liver cirrhosis' or exp cirrhosis/ or exp liver cirrhosis, mortality/ or liver disease:kw,ab
	**Specifics**
9	exp liver cirrhosis, alcoholic/
10	exp hepatitis B/exp or 'HBV' or exp hepatitis B, chronic/exp
11	exp hepatitis C/ or 'HCV' or exp hepatitis C, chronic/
12	exp metabolic disease/ or exp metabolic syndrome/ or exp non-alcoholic fatty liver disease/
13	exp autoimmune hepatitis/
14	9 or 10 or 11 or 12 or 13
15	3 and 7 and 8 and 14
16	remove duplicates from 15

Supplement Material 3. Cochrane Risk-of-Bias Tool for Non-Randomized Studies (ROBINS-I) adapted – risk of bias assessment

Signalling questions	Description	Response
**Bias due to confounding**		
1.1 Is there potential for confounding of the effect of alcohol in this study? **If <u>N/PN</u> to 1.1:** the study can be considered to be at low risk of bias due to confounding and no further signalling questions need be considered		
**If Y/PY to 1.1**: determine whether there is a need to assess time-varying confounding:		
1.2. Was the analysis based on splitting participants' follow up time according to alcohol groups? **If N/PN**, answer questions relating to baseline confounding (1.4 to 1.6) **If Y/PY**, go to question 1.3.		
1.3. Were changes in alcohol consumption likely to be related to factors that are prognostic for the outcome? **If N/PN**, answer questions relating to baseline confounding (1.4 to 1.6) **If Y/PY**, answer questions relating to both baseline and time-varying confounding (1.7 and 1.8)		
**Questions relating to baseline confounding only**		
1.4. Did the authors use an appropriate analysis method that controlled for all the important confounding domains?		
1.5. **If <u>Y/PY</u> to 1.4**: Were confounding domains that were controlled for measured validly and reliably by the variables available in this study?		
1.6. Did the authors control for any post-intervention variables that could have been affected by the alcohol?		
**Questions relating to baseline and time-varying confounding**		
1.7. Did the authors use an appropriate analysis method that controlled for all the important confounding domains and for time-varying confounding?		
1.8. **If <u>Y/PY</u> to 1.7**: Were confounding domains that were controlled for measured validly and reliably by the variables available in this study?		
**Risk of bias judgement**		
Quick Guide: ***Low:*** *age and/or sex and some variables from at least* ***<u>one</u>*** *other domain were included and measured reliability and validly* **+** *at least* **one** *time-varying variable* ***Moderate:*** *age and/or sex and some variables from at least* **one** *other domain were included and measured reliability and validly* **but** *no time-varying variable* ***Serious:*** *adjust for only one variable from any domain* ***Critical:*** *no adjustment*		
**Differences with table 1 of the original ROBINS-I guideline**		
Optional: What is the predicted direction of bias due to confounding?		
**Bias in selection of participants into the study**		
2.1. Was selection of participants into the study (or into the analysis) based on participant characteristics observed after the start of cohort? **If <u>N/PN</u> to 2.1:** go to 2.4 2.2. **If Y/PY to 2.1**: Were the variables that influenced selection likely to be associated with alcohol consumption? 2.3 **If Y/PY to 2.2**: Were the variables that influenced selection likely to be influenced by the outcome or a cause of the outcome?		
2.4. Does the start of follow-up coincide for most participants?		
2.5. **If Y/PY to 2.2 and 2.3, or N/PN to 2.4**: Were adjustment techniques used that are likely to correct for the presence of selection biases?		
**Risk of bias judgement**		

Quick Guide:<br>***Low:*** *no reason to think factors related to alcohol or cirrhosis influence non responding*<br>***Moderate:*** *selection into the study may have been related to alcohol and outcome;* ***AND*** *the authors used appropriate methods to adjust for (or address) the selection bias*<br>***Serious:*** *Selection was related (but not very strongly) to alcohol and outcome;* ***and*** *this could not be adjusted for in analyses*<br>***Critical:*** *Selection and/or retention was very strongly related to alcohol and outcome;* ***and*** *this could not be adjusted for in analyses*		
Optional: What is the predicted direction of bias due to selection of participants into the study?		
**Bias in classification of alcohol consumption**		
3.1 Were alcohol groups clearly defined?		
3.2 Was the information used to define alcohol groups recorded at the start of the study?		
3.3 Could classification of alcohol consumption have been affected by knowledge of the outcome or risk of the outcome?		
**Risk of bias judgement**		
Low: *Alcohol consumption groups are clearly defined, information recorded at the start of the study and was not affected by the knowledge of the outcome*<br>***Moderate:*** *Alcohol consumption groups are clearly defined but some information recorded retrospectively OR may have been affected by the knowledge of the outcome*<br>***Serious:*** *Alcohol consumption groups are clearly defined but some information recorded retrospectively AND may have been affected by the knowledge of the outcome*<br>***Critical:*** *Alcohol consumption groups are NOT clearly defined but some information recorded retrospectively AND may have been affected by the knowledge of the outcome*		
Optional: What is the predicted direction of bias due to measurement of alcohol?		
**Bias due to missing data**		
5.1 Were outcome data available for all, or nearly all, participants?		
5.2 Were participants excluded due to missing data on alcohol status?		
5.3 Were participants excluded due to missing data on other variables needed for the analysis?		
5.4 **If PN/N to 5.1, or Y/PY to 5.2 or 5.3**: Are the proportion of participants and reasons for missing data similar across alcohol groups?		
5.5 **If PN/N to 5.1, or Y/PY to 5.2 or 5.3**: Is there evidence that results were robust to the presence of missing data?		
**Risk of bias judgement**		
Low: *alcohol, main covariate, and outcome data were available for all, or nearly all participants* ***and*** *reasons for missing unlikely to be related to alcohol or outcome*<br>***Moderate:*** *between 10-20% missing data (including lost to follow-up) and reasons for missing unlikely to be related to alcohol or outcome*<br>***Serious:*** *more than 20% missing or between 10-20% missing data and reasons for missing* ***likely*** *to be related to alcohol or outcome*<br>***Critical:*** *(Unusual) There were critical differences between levels of alcohol or outcome in participants with missing data*		
Optional: What is the predicted direction of bias due to missing data?		
**Bias in measurement of outcomes**		
6.1 Could the outcome measure have been influenced by knowledge of the alcohol consumption ?		
6.2 Were outcome assessors aware of the alcohol consumption status of study participants?		

	6.3 Were the methods of outcome assessment comparable across alcohol groups?		
	6.4 Were any systematic errors in measurement of the outcome related to alcohol consumption?		
	**Risk of bias judgement**		
	Low: *the methods of outcome assessment were comparable across alcohol groups* **and** *unlikely to be influenced by knowledge of alcohol status, any error in measuring the outcome is unrelated to alcohol status.*<br>***Moderate:*** *the methods of outcome assessment were comparable across alcohol groups* **and** *minimally influenced by knowledge of alcohol status* ***(such as SR),*** *any error in measuring the outcome is only minimally related to alcohol status.*<br>***Serious:*** *the methods of outcome assessment were not comparable across alcohol groups* **and** *error in measuring the outcome is only minimally related to alcohol status.*<br>***Critical:*** *the methods of outcome assessment were so different that they cannot reasonably be compared across alcohol groups.*		
	Optional: What is the predicted direction of bias due to measurement of outcomes?		
**Bias in selection of the reported result**			
	Is the reported effect estimate likely to be selected, on the basis of the results, from...<br>7.1. ... multiple outcome *measurements* within the outcome domain?		
	7.2 ... multiple *analyses* of the exposure-outcome relationship?		
	7.3 ... different *subgroups*?		
	**Risk of bias judgement**		
	Low: *no possibility of reporting selected outcomes*<br>***Moderate:*** *multiple outcomes are reported with multiple analysis with no justification*<br>***Serious:*** *reported outcomes are not in line with objectives*<br>***Critical:*** *a clear selection of outcomes unrelated to objectives*		
	Optional: What is the predicted direction of bias due to selection of the reported result?		
**Overall bias**			
	**Risk of bias judgement**		
	Low: *low risk of bias for all domains*<br>***Moderate:*** *low or moderate risk of bias for all domains*<br>***Serious:*** *serious risk of bias in at least one domain*<br>***Critical:*** *critical risk of bias in at least one domain*		
	Optional: What is the overall predicted direction of bias for this outcome?		

Supplementary Material 4. Results of model selection

We tested five different models to evaluate the possibility of a non-linear dose-response relationship between alcohol consumption and the risk of liver cirrhosis in females and males. We obtained and compared the regression results, graphical representation and the log likelihood ratio test, AIC and BIC to select the best fitting model. The results were the following:

For females:

	Linear on the log scale regression	Quadratic regression	Restrictive cubic splines regression	Cubic polynomial regression	Multivariable fractional polynomial regression
**logLik:**	-112.6334	-54.79347	-50.95448	-53.39821	-54.79347
**deviance:**	225.2668	109.58694	101.90897	106.79641	109.58694
**AIC:**	229.2668	115.58694	109.90897	114.79641	115.58694
**BIC:**	232.7892	120.79995	116.76326	121.6507	120.79995
**AICc:**	229.5668	116.21852	111.02008	115.90752	116.21852

For males:

	Linear on the log scale regression	Quadratic regression	Restrictive cubic splines regression	Cubic polynomial regression	Multivariable fractional polynomial regression
**logLik:**	-179.0914	-171.915	-168.5328	-169.8623	-170.4032
**deviance:**	358.1828	343.83	337.0656	339.7245	340.8064
**AIC:**	362.1828	349.83	345.0656	347.7245	346.8064
**BIC:**	366.7637	356.66	354.1164	356.7752	353.6364
**AICc:**	362.3543	350.183	345.6717	348.3306	347.1593

The best fitting model for our data in females and in males was the restrictive cubic splines model, with the lowest AIC and BIC statistics. For this model, we set four knots at the corresponding fixed quantiles (5%, 35%, 65% and 95%) of the dose of alcohol consumed.

Supplementary Material 5. Final meta-regression model

We progressively introduced each variable, its corresponding interaction and quadratic interaction with the dose of alcohol consumption. The selected variables were sex, cause of liver cirrhosis, quality score and outcome. The quadratic model was selected to build the final regression model to avoid a complex model that would add difficulties to interpretation.

The most parsimonious model was selected with the following statistics: 15 degrees of freedom, AIC= 326.6481, BIC= 366.3140, AICc= 332.1027, logLik -148.3241. The final model was the following:

	**Estimate**	**Lower CI**	**Upper CI**	**pval**	**se**	**zval**
intercept	-0.5599*	-0.85715	-0.26265	0.00022	0.15166	-3.69179
dose	0.0626*	0.05511	0.07009	<.00001	0.00382	16.37875
dose^2	-0.00037*	-0.00044	-0.00029	<.00001	0.00004	-9.81849
Alcohol-related LC ‡	0.29493	-0.45869	1.04856	0.44306	0.38451	0.76704
HCV ‡	2.06029	0.04419	4.07639	0.04519	1.02864	2.00292
Alcohol-related LC + HCV ‡	2.29214*	0.5937	3.99057	0.00817	0.86657	2.64508
dose: male†	-0.0319*	-0.03987	-0.02393	<.00001	0.00407	-7.84141
(dose^2):male†	0.00027*	0.0002	0.00035	<.00001	0.00004	7.45262
dose: mortality ⫠	0.00842*	0.00051	0.01634	0.03707	0.00404	2.08496
(dose^2): mortality ⫠	-0.00009*	-0.00016	-0.00002	0.00924	0.00004	-2.60293
dose: Serious and critical quality score ɫ	0.01137*	0.00763	0.01512	<.00001	0.00191	5.95468
dose: alcohol-related LC‡	-0.01028	-0.02107	0.00052	0.06206	0.00551	-1.8659
dose: HCV LC‡	-0.02556*	-0.04103	-0.0101	0.0012	0.00789	-3.23969
(dose^2): alcohol-related LC‡	0.00013*	0.00003	0.00022	0.0076	0.00005	2.66936

*statistically significant; ‡ reference= all cause LC; †reference= female; ⫠ reference = morbidity; ɫ reference = moderate quality score
LC: liver cirrhosis; HCV: hepatitis C virus; US: United States; CI: confidence intervals; pval: *p* value; se: standard error; zval, z value

Variance Components:
sigma^2= 0.4941; sqrt= 0.7029   levels= 32

Test for Residual Heterogeneity:
QE(df = 104) = 897.9006, p-val < .0001

Test of Moderators (coefficients 2:14):
QM(df = 13) = 2773.1989, p-val < .0001

Supplementary Material 6. Meta-regression model by outcome

Morbidity studies:

	Estimate	Lower CI	Upper CI	pval	se	zval
intercept	-0.72453*	-1.34568	-0.10337	0.02225	0.31692	-2.28614
dose	0.06847*	0.05895	0.07799	<.00001	0.00486	14.09763
dose^2	-0.00037*	-0.00045	-0.00028	<.00001	0.00004	-8.20309
dose: male†	-0.03662*	-0.04733	-0.02592	<.00001	0.00546	-6.70336
(dose^2):male†	0.00026*	0.00016	0.00035	<.00001	0.00005	5.26154
dose: Serious and critical quality score ƚ	0.01364*	0.00852	0.01877	<.00001	0.00261	5.21913
dose: alcohol-related LC‡	-0.02042*	-0.03437	-0.00648	0.00411	0.00712	-2.86991
dose: HCV LC‡	0.02282	-0.002	0.04765	0.07157	0.01267	1.80185

*statistically significant; †reference= female; ƚ reference = moderate quality score; ‡ reference= all cause LC
LC: liver cirrhosis; HCV: hepatitis C virus; US: United States; CI: confidence intervals; pval: *p* value; se: standard error; zval, z value

Variance Components:
sigma^2= 1.26523 sqrt=1.12483 levels=15

Test for Residual Heterogeneity:
QE(df = 38) = 276.35288, p-val < .00001

Test of Moderators (coefficients 2:8):
QM(df = 7) = 1499.42477, p-val < .00001

Mortality studies:

	Estimate	Lower CI	Upper CI	pval	se	zval
intercept	-0.37*	-0.641	-0.099	0.00745	0.13827	-2.67595
dose	0.07453*	0.05982	0.08924	<.00001	0.0075	9.93097
dose^2	-0.00054*	-0.00068	-0.0004	<.00001	0.00007	-7.55718
dose: male†	-0.03587*	-0.04932	-0.02242	<.00001	0.00686	-5.22642
(dose^2):male†	0.00036*	0.00024	0.00049	<.00001	0.00006	5.59214
dose: Serious and critical quality score ƚ	0.00309	-0.00823	0.0144	0.59277	0.00577	0.53483
(dose^2): Serious and critical quality score ƚ	0.00011*	0.00001	0.00021	0.02936	0.00005	2.17866
dose: alcohol-related LC‡	0.00034	-0.0049	0.00557	0.89989	0.00267	0.1258

*statistically significant; †reference= female; ƚ reference = moderate quality score; ‡ reference= all cause LC
LC: liver cirrhosis; HCV: hepatitis C virus; US: United States; CI: confidence intervals; pval: *p* value; se: standard error; zval, z value

Variance Components:
sigma^2= 0.23258; sqrt= 0.48227; levels=17

Test for Residual Heterogeneity:
QE(df = 64) = 625.16691, p-val < .00001

Test of Moderators (coefficients 2:8):
QM(df = 7) = 1276.93005, p-val < .00001

Supplementary Material 7. Meta-regression model by sex

Female studies:

	**Estimate**	**Lower CI**	**Upper CI**	**pval**	**se**	**zval**
intercept	-0.3516	-1.31978	0.61658	0.47661	0.49398	-0.71177
dose	0.06856*	0.05898	0.07814	<.00001	0.00489	14.02383
dose^2	-0.00031*	-0.00042	-0.0002	<.00001	0.00005	-5.72759
Mortality ⫠	0.61271	-0.37388	1.5993	0.22352	0.50337	1.21721
Serious and critical quality score ⸸	-0.67654	-1.72066	0.36758	0.2041	0.53272	-1.26996
dose: mortality ⫠	-0.00888	-0.02718	0.00942	0.34157	0.00934	-0.95106
(dose^2): mortality ⫠	-0.00002	-0.00017	0.00012	0.73878	0.00007	-0.33347
dose: Serious and critical quality score ⸸	0.00379	-0.00735	0.01492	0.50498	0.00568	0.66667
dose: alcohol-related LC‡	0.01159*	0.00319	0.01999	0.00683	0.00429	2.70503

*statistically significant; ⫠ reference = morbidity; ⸸ reference = moderate quality score; ‡ reference= all cause LC
LC: liver cirrhosis; HCV: hepatitis C virus; US: United States; CI: confidence intervals; pval: *p* value; se: standard error; zval, z value

Variance Components:
sigma^2= 0.72648, sqrt=0.85234; levels= 13

Test for Residual Heterogeneity:
QE(df = 35) = 206.06421, p-val < .00001

Test of Moderators (coefficients 2:9):
QM(df = 8) = 638.10179, p-val < .00001

Male studies:

	**Estimate**	**Lower CI**	**Upper CI**	**pval**	**se**	**zval**
intercept	-0.70022	-1.11433	-0.28612	0.00092	0.21128	-3.31417
dose	0.03251	0.02755	0.03746	<.00001	0.00253	12.85333
dose^2	-0.00012	-0.00017	-0.00007	0.00002	0.00003	-4.32443
Alcohol-related LC ‡	0.57773	-0.51882	1.67427	0.30178	0.55947	1.03263
HCV ‡	2.04801	-0.02547	4.12148	0.05288	1.05791	1.93589
Alcohol-related LC + HCV ‡	2.01812	0.25045	3.78579	0.02524	0.90189	2.23766
dose: mortality ⫠	0.01164	0.00264	0.02063	0.01122	0.00459	2.53589
(dose^2): mortality ⫠	-0.00009	-0.00017	-0.00001	0.02363	0.00004	-2.26309
dose: Serious and critical quality score ⸸	0.01644	0.01206	0.02081	<.00001	0.00223	7.35676
dose: alcohol-related LC‡	-0.02218	-0.03462	-0.00973	0.00048	0.00635	-3.49272
dose: HCV LC‡	-0.02753	-0.04309	-0.01196	0.00053	0.00794	-3.46681
(dose^2): alcohol-related LC‡	0.00017	0.00007	0.00027	0.00112	0.00005	3.2578

*statistically significant; ‡ reference= all cause LC; ⫠ reference = morbidity; ⸸ reference = moderate quality score
LC: liver cirrhosis; HCV: hepatitis C virus; US: United States; CI: confidence intervals; pval: *p* value; se: standard error; zval, z value

Variance Components:
sigma^2= 0.53283; sqrt=0.72995; levels=19

Test for Residual Heterogeneity:
QE(df = 62) = 522.25827, p-val < .00001

Test of Moderators (coefficients 2:12):
QM(df = 11) = 2163.62526, p-val < .00001

Supplement Material 8. Sensitivity analysis

We conducted an analysis excluding the study of Liu and colleges. The results obtained for the meta-regression model were the following:

	**Estimate**	**Lower CI**	**Upper CI**	**pval**	**se**	**zval**
intercept	-0.51024*	-0.8076	-0.21288	0.00077	0.15172	-3.36311
dose	0.0599*	0.05153	0.06828	<.00001	0.00427	14.02091
dose^2	-0.00035*	-0.00042	-0.00027	<.00001	0.00004	-8.68316
Alcohol-related LC ‡	0.25072	-0.49008	0.99152	0.50711	0.37797	0.66334
HCV ‡	2.0241*	0.0269	4.0213	0.04699	1.019	1.98636
Alcohol-related LC + HCV ‡	2.25665*	0.58073	3.93256	0.00831	0.85507	2.63912
dose: male†	-0.02948*	-0.03819	-0.02077	<.00001	0.00444	-6.63423
(dose^2):male†	0.00026*	0.00018	0.00033	<.00001	0.00004	6.57508
dose: mortality ⊥	0.00942*	0.00132	0.01751	0.02259	0.00413	2.28025
(dose^2): mortality ⊥	-0.0001*	-0.00017	-0.00003	0.0056	0.00004	-2.77049
dose: Serious and critical quality score ł	0.01127*	0.0075	0.01504	<.00001	0.00192	5.86324
dose: alcohol-related LC‡	-0.01032	-0.0212	0.00056	0.06312	0.00555	-1.85837
dose: HCV LC‡	-0.02561*	-0.04108	-0.01015	0.00117	0.00789	-3.24568
(dose^2): alcohol-related LC‡	0.00013*	0.00003	0.00023	0.00757	0.00005	2.67081

*statistically significant; ‡ reference= all cause LC; †reference= female; ⊥ reference = morbidity; ł reference = moderate quality score
LC: liver cirrhosis; HCV: hepatitis C virus; US: United States; CI: confidence intervals; pval: *p* value; se: standard error; zval, z value
